# Nanoforms of essential metals: from hormetic phytoeffects to agricultural potential

**DOI:** 10.1093/jxb/erab547

**Published:** 2021-12-18

**Authors:** Zsuzsanna Kolbert, Réka Szőllősi, Andrea Rónavári, Árpád Molnár

**Affiliations:** Department of Plant Biology University of Szeged, Közép fasor 52, Szeged H6726, Hungary; Department of Plant Biology University of Szeged, Közép fasor 52, Szeged H6726, Hungary; Department of Applied and Environmental Chemistry, University of Szeged, Rerrich Béla tér 1, Szeged H6720, Hungary; Department of Plant Biology University of Szeged, Közép fasor 52, Szeged H6726, Hungary; Hasselt University, Belgium

**Keywords:** Hormesis, nanofertilization, nanometals, nanopriming, nutrient deficiency, omics

## Abstract

Vital plant functions require at least six metals (copper, iron, molybdenum, manganese, zinc, and nickel), which function as enzyme cofactors or inducers. In recent decades, rapidly evolving nanotechnology has created nanoforms of essential metals and their compounds (e.g. nZnO, nFe_2_O_3_) with a number of favourable properties over the bulk materials. The effects of nanometals on plants are concentration-dependent (hormesis) but also depend on the properties of the nanometals, the plant species, and the treatment conditions. Here, we review studies examining plant responses to essential nanometal treatments using a (multi)omics approach and emphasize the importance of gaining a holistic view of the diverse effects. Furthermore, we discuss the beneficial effects of essential nanometals on plants, which provide the basis for their application in crop production as, for example, nanopriming or nanostimulator agents, or nanofertilizers. As lower environmental impact and increased yield can be achieved by the application of essential nanometals, they support sustainable agriculture. Recent studies have actively examined the utilization of green-synthesized metal nanoparticles, which perfectly fit into the environmentally friendly trend of future agriculture. Further knowledge is required before essential nanometals can be safely applied in agriculture, but it is a promising direction that is timely to investigate.

## Teaser

Despite the diversity of nanometal-induced hormetic effects, the application of nanometals in sustainable agriculture is promising, but should be preceded by detailed examination of plant responses using (multi)omics.

## Introduction

Essential elements are indispensable for the vegetative growth and reproduction of plants and are not replaceable by another element. These elements are directly involved in the metabolism forming structural or functional components of plant cells. Moreover, inadequate availability of essential elements causes damage to the plant. These criteria are met by certain metals, such as copper (Cu), iron (Fe), molybdenum (Mo), manganese (Mn), zinc (Zn), and nickel (Ni), which are essential trace components of plant structures (Barker, 2015).

Land plants have specific transport systems to absorb essential metal ions effectively from the environment, appropriately distribute them in plant organs and tissues, and sequester them within cells (Andresen *et al.*, 2018). The transport of trace metal ions across membranes requires energy derived directly from ATP hydrolysis (primary active transport) or provided by the metal transporter due to the symport or antiport of other ions (e.g. H^+^; secondary active transport). Primary active transport of essential metal ions is mediated by P2A and P1B-type (also called CPx-type) ATPases (Williams and Mills, 2005), while glutathione- or phytochelatin-bounded metal ion ligands are transported by ABC proteins (Dahuja *et al.*, 2021). Most ABC transporters are not specifically involved in metal translocation but are involved in other transport routes (e.g. malate transport, indole-acetic acid transport; Dahuja *et al.*, 2021). Secondary active transport systems associated with metal uptake into the cytoplasm and intracellular translocation are natural resistance-associated macrophage proteins (NRAMPs), ZRT/IRT-like proteins (ZIPs), and cation diffusion facilitators (CDFs) (Hall and Williams, 2003; Lira-Morales *et al.*, 2019). The latter group of transporters are mainly embedded in the vacuolar or Golgi membranes and function as metal–proton antiporters. Additional types of transporters involved in metal translocation are cation/proton exchangers (CAXs) found in the tonoplast (Pittman and Hirschi, 2016). Additionally, endocytosis and exocytosis may be involved in metal uptake and export from the cytoplasm (Andresen *et al.*, 2018).

In plant cells, essential metals can be present in diverse biologically active forms such as hydrated ions, cofactors in proteins, nucleic acid-bound forms, and forms associated with amino acids, glutathione, nicotinamine, citrate, and malate (Juárez-Maldonado *et al.*, 2018). Among their chemical properties, redox activity has significant relevance in influencing the specific biological function of the essential metals. Due to their different oxidation states, Cu and Fe catalyse oxido-reduction reactions in active centres of enzymes, and thus they are involved in electron transport and cellular redox control.

As the cofactor of plastocyanin, Cu has a unique role in photosynthetic electron transport (Droppa and Horváth, 1990). Cu is involved also in the mitochondrial electron transport chain as a cofactor of cytochrome oxidase, which contains also Fe (in the form of haem) as a supporter of its function. Cu also has a role in reactive oxygen species (ROS) detoxification, since it activates Cu/Zn superoxide dismutase (SOD) isoenzymes and ascorbate oxidase (Alscher *et al.*, 2002; De Tullio *et al.*, 2013). There are several other Cu-dependent oxidases, such as laccase, polyphenol oxidase, and amine oxidase, all of which are involved in wound healing and thus in plants’ defence against pathogens (Li *et al.*, 2019). Moreover, Cu is found in the active site of ethylene receptors and thus has a relevant role in perception of the hormone ethylene in plant cells (Li *et al.*, 2019; Schott-Verdugo *et al.*, 2019).

The antioxidant enzyme SOD has an isoform that functions in the presence of Fe. The biological roles of Cu/ZnSODs and FeSODs are different, since FeSODs control the level of ROS as signal molecules (Alscher *et al.*, 2002). Alternative oxidase, involved in respiration, is also a protein that binds haem-Fe. Furthermore, there are multi-haem proteins belonging to the P450 superfamily. These enzymes control redox reactions and convert various types of secondary metabolites, such as amines, fatty acids, and steroids. In addition, multiple steps of lignin biosynthesis involve the action of P450 superfamily enzymes. Fe is involved directly in the light reactions of photosynthesis, since it is a substituent of cytochrome *b*_559_ in the PSII reaction centre and it forms Fe_4_S_4_ clusters in PSI reaction centres. Additionally, the cytochrome *b*_6_*f* complex contains both haem and Fe-S clusters. In the respiratory electron transport chain, Fe-S clusters can be found in complex I and II, and in the cytochrome *bc*_1_ complex. Nitrate and sulfate metabolisms also require available Fe, since the enzymes nitrate reductase (NR), nitrite reductase (NiR), and sulfite oxidase (SO) contain haem-Fe (Vigani and Murgia, 2018).

NR and SO enzymes contain also Mo in the form of an organic cofactor, molybdopterin, which is involved in substrate binding and reduction. Aldehyde oxidases (acting in abscisic acid and indoleacetic acid biosynthesis) and xanthine oxidase are additional enzymes that function in the presence of Mo, and these enzymes contain a Fe_2_S_2_ cluster as well (Mendel and Hänsch, 2002).

The major role of Mn in plants is related to the fact that it is a central substituent of the water-splitting complex in the PSII reaction centre. Furthermore, ROS-detoxifying MnSODs are present in mitochondria and in peroxisomes. Mn is also bounded by germin and germin-like proteins, which are located in the cell walls and catalyse the production of hydrogen peroxide (H_2_O_2_) from oxalate. H_2_O_2_ contributes to pathogen defence as a signal, as an antimicrobial agent, and as an inducer of lignification. There are several enzymes (e.g. decarboxylases and dehydrogenases in the tricarboxylic acid cycle; phenylalanine ammonia lyase in the shikimic acid pathway of secondary metabolite synthesis) that do not bind Mn but are activated in the presence of Mn (Schmidt and Husted, 2019).

The biological functions and roles of Zn differ from those of Cu and Fe, since Zn is not a redox-active metal. Zn does not catalyse redox reactions but it acts as a Lewis base at the active sites of enzymes or is a structural component of proteins. As a cofactor, Zn is present in all six enzyme classes. Carbonic anhydrase is a very important Zn-dependent plant enzyme, which catalyses the interconversion of carbon dioxide and bicarbonate. Besides Cu, Fe, and Mn, Zn is also involved in ROS detoxification due to its binding to the active site of Cu/ZnSOD (Castillo-Gonzales *et al*., 2018). There are multiple phosphatase enzymes that contain Zn. The active sites of alkaline phosphatases contain one magnesium (Mg) ion (Mg^2+^) and two Zn^2+^, while purple acid phosphatases contain an active site with one Fe^3+^ and one Zn^2+^ bound. Due to its regulatory role in phosphatases, Zn is involved in phosphate nutrition (Olczak *et al.*, 2003). Another interesting enzyme is *S*-nitrosoglutathione reductase, which regulates nitric oxide metabolism and signalling, and which contains catalytic and structural Zn ions (Lindermayr, 2018). Zinc-finger transcription factors bind Zn and undergo conformational changes leading to altered DNA-binding activity. This means that Zn controls gene expression via the regulation of non-protein transcription factors (Takatsuji, 1998).

Ni is a very special metal, since the only known plant enzyme that requires Ni for its function is urease. Urea is the end-product of protein and ureide degradation, and urease detoxifies urea, catalysing the recycling of nitrogen (N) (Fabiano *et al.*, 2015).

Collectively, plant cells contain at least six types of metals that are essential for basic life functions. Essential metals are part of the metallome, which can be defined as the sum of the organic and inorganic metal forms in the cell, changes in which affect the physiological processes of plants (Singh and Verma, 2018).

## Size matters: beneficial properties of nanoforms

Nanotechnology has become a dynamically emerging industry, with recent manufacturing developments in science and technology leading to the generation of materials of different shapes and sizes, with nanosized materials being among them. These advancements provide the basis for further design to create unique properties targeted toward specific applications (Khot *et al.*, 2012).

**Fig. 1.**
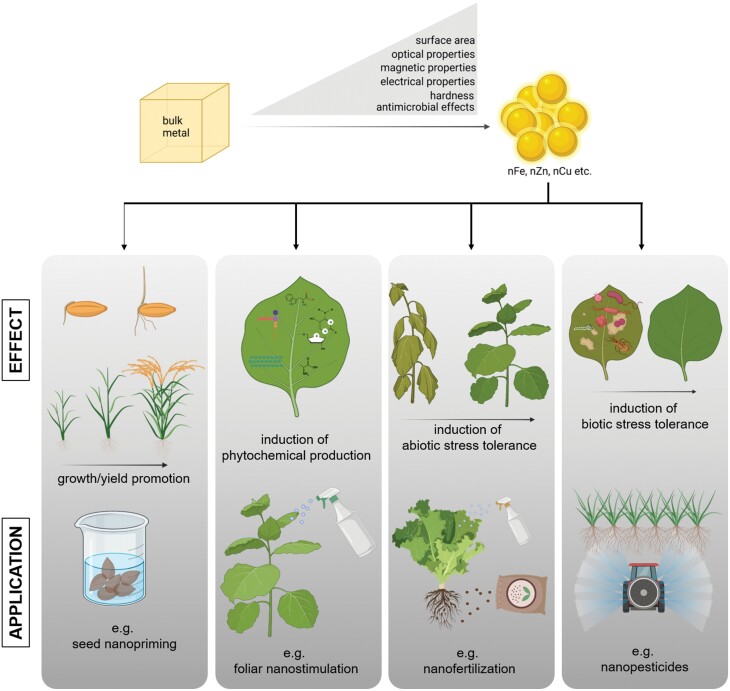
Unique properties of nanometals compared with the bulk form, and the phytoeffects and application of nanometals in plant cultivation. The nanoforms of essential metals possess greater surface area and hardness, specific optical, magnetic, and electrical properties, and show antimicrobial effects. Essential nanometals promote growth/yield and induce the synthesis of phytochemicals and protection against abiotic and biotic stressors. The positive effects of essential nanometals on the physiology of plants can be utilized during agricultural approaches such as nanopriming, nanostimulation, nanofertilization, and nanopesticide application. For further details refer to the text.

These nanosized materials, the so-called nanomaterials and nanoparticles (NPs), which have sizes typically below 100 nm, refer to a colloidal particle system that has received much attention in relation to chemical and biological applications because of their unique structure and tunable physico-chemical and biological properties, such as electrical and thermal conductivity, light absorbance, and catalytic activity, as well as antimicrobial activity, resulting in greater performance compared with their bulk counterparts (Fig. 1). These versatile properties can change as a function of the size and shape of the material. The dissolution, surface reactivity, and aggregation states of nanomaterials play key roles in their fate, lifetime, behaviour, and interactions in environments, often with global complex effects.

Metals and metal oxides in nanosized form can exist naturally in the environment; they are generated in nature either by biological species or through anthropogenic activities. They can also be manufactured (the so-called engineered nanomaterials) by several routes including chemical, physical, and biological methods (e.g. chemical reduction, photochemical methods, microwave processing, laser ablation, and grinding). The synthesis of NPs by chemical and physical means is more frequent, and the use of toxic and expensive chemicals in these processes limits their applications in daily life, human health, and agriculture-related applications. Therefore, the biogenic approaches, which utilize microbial or plant sources, have emerged recently as novel and potential methods for producing NPs. The rapid development of the synthesis procedures brought a significant increase in the number of nanomaterials with desired properties being manufactured (Gebre and Sendeku, 2019).

Nanometals have a metal core composed of inorganic metal or metal oxide that is usually covered with a thin oxide shell. They can be produced with different morphologies, such as NPs, nanofibers, nanowires, nanotubes, or nanosheets, possessing different kinds of promising features, such as size-dependent qualities, high surface-to-volume ratio, specific surface plasma resonance, increased optical and magnetic properties, and enhanced antimicrobial activity (Jagadish *et al.*, 2018). Due to their reduced molecular size and increased surface area, and also because of changed interactions between molecules, nanomaterials have attributes that may be novel and often differ significantly from those of the bulk material, and which are not characteristics on the micro- or macroscopic scale, or in ionic form.

It is well known that the physico-chemical features of NPs are determined by a number of factors, such as NP size and morphology, or by the nature of the capping materials covering the particle surface (Rónavári *et al.*, 2017). Therefore, proper selection of synthesis methods including adequate reducing and stabilizing agents is crucial to achieve the desired particle properties. As metal NPs are highly reactive, and rapidly react with oxygen or biomolecules in living systems, surface modification is frequently applied to prevent aggregation. These surface adjustments can be achieved by the use of stabilizing agents, coating, or functionalizing (e.g. with oleic acid, sodium citrate, polymers, or biomolecules), promoting the dispersion of NPs and uniform particle size distribution. Various biomolecules (e.g. proteins, polysaccharides, and peptides) have been also used in the biosynthesis of NPs. These molecules interact with the NPs and are able to adsorb on to NP surfaces, establishing the so-called biomolecular corona (Bélteky *et al.*, 2021). This corona serves as a kind of organic complex on the surface of the NP that can improve the NP stability; moreover, it significantly determines and could improve the properties, behaviour, and evoked responses of NPs in the environment and living systems (e.g. enhanced particle release, less toxicity). Thus, the characteristics and fate of metal NPs can be designed and controlled on demand by using different biomolecules during their synthesis, as a function of the specific purpose.

## Behaviour of essential nanometals on root/leaf surfaces and in plant cells

The uptake and transport of nanometals is a complex and in some cases contradictory process. To enter plant tissues, NPs need to breach multiple barriers that protect cells from hazards. These protective layers vary across species, tissues, and environmental conditions (Schwab *et al.*, 2016). In case of the root system, the first line of defence is a layer of rhizosphere bacteria and fungi. Symbiotic microenvironments influence the mobility of nanometals in soil, affecting their uptake. For example, in ZnO and CuO NP-treated mung bean, *Pseudomonas chlororaphis* O6 affected the uptake of essential metals (Dimkpa *et al.*, 2015). It is known that mycorrhizae can improve heavy metal tolerance in plants and increase the soil porosity in response to FeO NPs (Feng *et al.*, 2013). The second physiological barrier near plant roots consists of mucilage and exudates (M/E) excreted into the soil. These biomolecules have a role in sensing and protecting the root system on the outer cell surface (McNear, 2013). M/E can reversibly absorb various NPs near the root cap and root hairs (Aubert *et al.*, 2012; Zhu *et al.*, 2012; Lee *et al.*, 2013). M/E has a role in acidifying the root microenvironment, which could intensify the dissolution of nanometals (Lv *et al.*, 2015). It is often reported that nanometals in the roots are smaller in diameter than those in the treatment solution, and in some cases they are completely dissolved (Lv *et al.*, 2015). The effectiveness of M/E in alleviating NP stress is variable; it is more pronounced in the case of small and positively charged nanomaterials (Zhu *et al.*, 2012). The aerial plant parts are protected by a lipophilic cuticle barrier that consists of epicuticular wax crystals, which create a robust and almost impenetrable layer (Albersheim, 2011). It is highly unlikely that hydrophilic NPs can cross this barrier; however, thin permeable regions with uptake or secretory functions are suggested to function as uptake sites. Beyond the root system, nanomaterials can enter the plant body via the opened stomata, where the water solubility of ZnO and MgO resulted in better translocation to the roots (Wang *et al.*, 2013). Plant cell walls are protective layers of fibres and other composites with relatively continuous porosity (Schwab *et al.*, 2016). The pore size of the cell walls (defined as the space between the cell wall components within the wall matrix) is mostly between 0.6 nm and 4.8 nm (Eichert and Goldbach, 2008; Kurczyńska *et al.*, 2021), which is favourable for extremely small neutrally charged NPs. There is experimental evidence that nanometals such as Fe_3_O_4_-NP (18 nm) or ZnO NP (8 nm) enter the cytoplasm (Molnár *et al.*, 2020; Iannone *et al.*, 2021), which can be made possible by the NP-induced modification of the chemical (macromolecule composition) and physical (pore size) properties of the cell wall, as suggested by Kurczyńska *et al.* (2021). The Casparian strip is the major barrier to apoplastic transport, and it has been suggested that NPs can enter the pericycle via the disruption of this barrier in early lateral root development (Lv *et al.*, 2015). Metallic NPs are usually charged, making them unable to cross the plasma membrane by diffusion. However, it is documented clearly that metal NPs enter the cells (reviewed by Bhirde *et al.*, 2011; Lv *et al.*, 2019), suggesting possible uptake mechanisms. It has been documented that NP endocytosis can occur via multiple pathways (Onelli *et al.*, 2008; Palocci *et al.*, 2017); however, results with essential nanometals are scarce. Moreover, NP binding to carrier proteins or ion channels has also been proposed as an NP uptake mechanism (Brandenberger *et al.*, 2010; Shukla *et al.*, 2016). Symplastic transport between cells via plasmodesmata, which are 20–50 nm in diameter, has also been proposed. Small particles of ~3 nm can be transported via plasmodesmata between cells (Dietz and Herth, 2011; recently reviewed by Kurczyńska *et al.*, 2021). Furthermore, NPs are assumed to influence the size exclusion limit of plasmodesmata, allowing bigger NPs to travel through them (Kurczyńska *et al.*, 2021). Interorganellar translocation of NPs has been documented in various plant species. For instance, FeO NPs were taken up by pumpkin roots and transferred to the leaves via the xylem (Zhu *et al.*, 2008; reviewed by Peng *et al.*, 2020).

In the past, the detection of nanomaterials in plant tissues provided contradictory results, partly due to the different detection techniques, nanomaterial transformation processes, nanomaterial sizes and properties, and experimental designs used in studies (Montes *et al.*, 2017; Wojcieszek *et al.*, 2020). Nowadays, numerous methods are being used in nanomaterials research, which will be summarized briefly below. Synchrotron-based X-ray fluorescence mapping and X-ray absorption spectroscopy is one of the newest methods in this field. It can provide details about the elemental composition, location, and chemical properties of nanomaterials. Due to the nature of this method, it can detect biotransformation of nanomaterials, making it an excellent tool to visualize the behaviour of NPs in live tissues (Castillo-Michel *et al.*, 2017). Inductively coupled plasma-mass spectrometry (ICP-MS) is able to detect metals in tissues in the extremely low ng l^–1^ range. The technique is limited due to the sample preparation method, since it can measure metal content only from acid-decomposed samples. A new technique, single-particle ICP-MS, has been developed, which is able to measure dissolved particles, and can measure each NP individually (Wojcieszek *et al.*, 2020). It is able to detect NP alterations, which is crucial information in samples. Imaging methods used in visualizing the uptake and localization of nanomaterials in tissues include transmission and scanning electron microscopy (TEM and SEM), matrix-assisted laser desorption ionization (MALDI), and laser ablation (LA) ICP-MS (Montes *et al.*, 2017; Wojcieszek *et al.*, 2020). The most prevalent technique is TEM, which can provide information about the localization, size, and aggregation of nanomaterials in cells. It has a high spatial resolution (<1 nm); however, the interpretation of the data requires multiple control samples. To evaluate the behaviour of nanomaterials in tissues, a combi­nation of various methods is recommended to obtain detailed results.

It has been well documented that the synthesized engineered form of NPs changes when they are released into the environment. Nanomaterials are transformed by natural organic matter in ways including dissolution, chelation, precipitation, and reactions with various functional groups (Maurer-Jones *et al.*, 2013). Additionally, plant biochemical pathways have a tendency to modify internalized nanomaterials. In aqueous solutions, the stability of nanomaterials is influenced heavily by dissolution and aggregation, which reduce surface size. Ion release from metal NPs is considered to be a quick process in the case of Cu (Conway *et al.*, 2015) and Zn (David *et al.*, 2012) NPs. Dissolution of Zn ions from ZnO NPs is hypothesized to be one of the main pathways of NP toxicity (Kouhi *et al.*, 2014). In realistic conditions, natural organic matter can limit NP aggregation rates, thus stabilizing the solution (Liu *et al.*, 2012). Different soil microenvironments also transform nanomaterials. In clay soils, reducing conditions are present, whereas in sandy soils oxidative transformations are favoured (Zhang *et al.* 2020). On leaf surfaces in the presence of oxygen, redox-active metals such as Cu NPs can receive an oxide coating (Tani *et al.*, 2018). Additionally, ZnO NPs have been reported to be transformed into other inorganic (Zn-nitrate, Zn-phosphate) and organic (Zn-citrate) forms by desert plants (De la Rosa *et al.*, 2011). Biomolecular coatings on NPs called coronas (as described above) have a significant effect on NP properties. The chemical composition of the corona can influence the uptake (Francia *et al.*, 2019), dissolution (Collin *et al.*, 2016), and spatial distribution of NPs. In the case of pumpkin xylem fluid, the corona mainly consists of selectively bound proteins on the surface of CuO NPs (Borgatta *et al.*, 2021). It is hypothesized that the positively charged NP surface interacts with partially negatively charged functional groups in proteins. Interestingly, it has been reported that metal ions can transform into NPs that are naturally in certain plant tissues (Kuppusamy *et al.*, 2016).

Taken together, the uptake, translocation, and transformation of (essential) nanometals in biological systems is still not understood fully. Analysing the transformed NPs in plant tissues has several methodological difficulties; however, detailed knowledge of these processes is of paramount importance.

## The hormetic effects of essential nanometals in plants: an omics approach

**Fig. 2.**
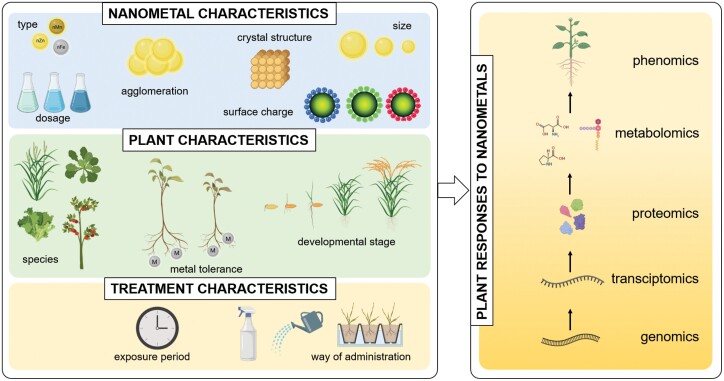
Factors determining plant responses to nanometals and the different levels at which plant responses can be examined. Among the characteristics of nanometals, their type, dosage, size, agglomeration, crystal structure, and surface charge are the most important determining factors. The response to essential nanometals also depends on the species, metal tolerance capacity, and developmental stage of the plant. Additionally, the method of application (e.g. foliar spray, irrigation, hydroponics) and the period of exposure to the nanometal also influence its effect. Plants show multi-level responses to nanometal exposure. Genomics, transcriptomics, proteomics, metabolomics, and phenomics studies provide a holistic view about the complex effects of essential nanometals on plants.

The effect of essential nanometals on plants depends on various factors, including the characteristics of the nanometal and also of the interacting plant partner (Fig. 2). The physico-chemical properties of nanometals, such as their hydrodynamic size, morphology, crystal structure, agglomeration, surface charge, and modification, influence the degree of their effects on plants. From the plant side, the species dependence of the effects of nanometals has been described, and the metal accumulation capacity and tolerance of the plant species also determine the effects of the nanometal. The evoked response to the presence of nanometals may also depend on the developmental stage of the plant. From the methodological point of view, the duration of exposure to nanometals and the way in which they are administered also influence their effects on plants (Fig. 2).

**Fig. 3.**
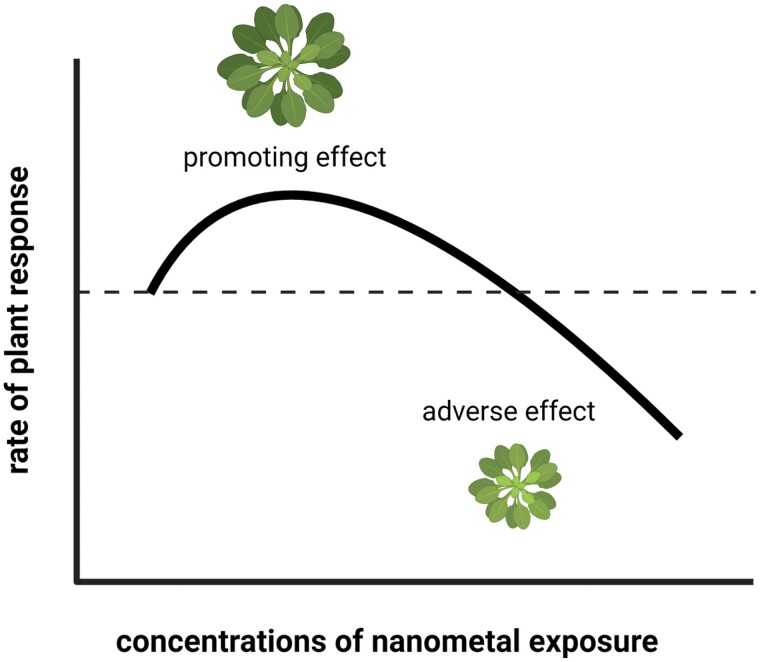
Hormetic effect of increasing doses of essential nanometals on plants. At lower concentrations, nanometals promote plant biomass production and physiological processes, whereas higher nanometal concentrations exert inhibitory effects.

Literature data agree that plants respond to the presence of essential nanometals in a concentration-dependent way, which—using a toxicological term—is called hormesis (Agathokleous *et al.* 2019b). The response of plants to metallic micronutrients and nanometals depends on the concentration in such a way that stimulation occurs in a lower concentration range, whereas higher doses exert adverse effects on plants (Fig. 3).

In their work evaluating the relevant literature, Agathokleous *et al.* (2019a) revealed that the extent of the hormetic plant responses to nanomaterials (not only to nanometals) depends on the size and type of the nanomaterial, since different nanomaterials caused different hormetic effects when applied at the same concentration under the same experimental conditions. The amplitude of the hormetic response proved to depend also on whether the nanomaterial contains essential or non-essential nutrients. Based on the analysis, element release from the NPs does not seem to be the major mechanism responsible for the stimulatory effect of low NP concentrations (Agathokleous *et al.* 2019a).

According to the holistic view, plant responses to essential nanometals are the sum of changes at the genome, transcriptome, proteome, metabolome, ionome, and phenome levels (Fig. 2). Few studies have evaluated transcriptome-level changes in plants treated with essential nanometals (Table 1). For example, Landa *et al.* (2017) observed that exposure to CuO NPs (10 mg l^–1^) altered the expression of 922 genes (mainly involved in stress responses and Cu binding/transport), whereas Cu ion treatment resulted in the up-regulation of 482 genes in *Arabidopsis thaliana* L. roots. Moreover, the similar transcription profiles of NP- and ion-exposed plants indicates that the release of Cu^2+^ ions from CuO NPs can be considered as the main contributor to phytotoxicity. More recently, 2270 and 4264 genes were found to be differentially expressed in response to exposure to 100 mg l^–1^ and 1000 mg l^–1^ CuO-NPs, respectively, in lettuce. The affected genes belonged to the ABC transporter family and heavy metal-associated isoprenylated plant proteins (HIPPs), which are involved in endocytosis, metal ion binding, and transport (Xiong *et al.*, 2021). Moreover, genes associated with cell wall biogenesis, antioxidant processes, and photosynthesis were also affected by CuO NP treatment of lettuce (Xiong *et al.*, 2021). Using metabolomics platforms, 65 metabolites, including jasmonates, scopoletin, and glucosinolate derivatives, were detected in leaves of nano CuO-treated Arabidopsis (Chavez Soria *et al.*, 2019). Targeted analysis of the jasmonic acid (JA) pathway revealed that CuO NP up-regulates JA precursors and down-regulates JA levels, indicating the involvement of JA-associated defence signalling in plant responses to CuO NPs. As for proteome-level changes induced by CuO NPs, the abundance of proteins involved in glycolysis and the tricarboxylic acid cycle increased in stress-tolerant wheat (*Triticum aestivum* L.) varieties, while photosynthesis- and tetrapyrrole synthesis-related proteins were down-regulated upon nano CuO exposure (Yasmeen *et al.*, 2018). Using a gel-free proteomic approach in grains of CuO NP-exposed wheat, elevated levels of proteins associated with starch degradation and glycolysis have been detected (Yasmeen *et al.*, 2017). Multiple studies revealed that nano ZnO extensively modifies the gene expression profiles of Arabidopsis and tomato (*Solanum lycopersicum* L.), mainly up-regulating biotic- and abiotic stress-related genes (Landa *et al.*, 2012; Wan *et al.*, 2019; Sun *et al*; 2020). Furthermore, ZnO NPs induced changes in the secondary metabolism and increased the amino acid and sugar contents in the leaves and roots of tomato (Sun *et al.*, 2020). In response to nano ZnO exposure of soybean (*Glycine max* L.), 298 root proteins showed specific modifications, whereas in the shoot the expression of only 36 proteins was altered exclusively by nano ZnO (Hossain *et al.*, 2016). Using gel-free proteomics, Yasmeen *et al.* (2016) found that Fe NP treatment affected proteins mainly associated with photosynthetic processes in wheat. Moreover, an enhanced abundance of light-reaction-related proteins was associated with salt tolerance. In Fe NP-exposed wheat seeds, Yasmeen *et al.* (2017) described the accumulation of proteins involved in starch degradation, glycolysis, and the tricarboxylic acid cycle. In a recent metabolomics study, 21 and 53 dysregulated metabolites were detected in molybdenum trioxide (MoO_3_) NP-treated corn (*Zea mays* L.) and wheat leaves, respectively. Notably, more metabolic pathways were affected by the NPs in wheat than in corn. The untargeted analyses were completed by the analysis of individual groups of metabolites and, for example, asparagine, fructose, reduced glutathione, and mannose were found to be modulated by MoO_3_ NPs in both corn and wheat (Huang *et al.*, 2021).

**Table 1.** Transcriptomic, proteomic, and metabolomic studies in plants treated with essential nanometals

Essential nanometal	Plant species	Nanometal treatment	Approach/technology	Most relevant effects	Reference
Transcriptomics					
nCuO	*Arabidopsis thaliana* (Col-0), root	10 mg l^–1^ nCuO (<50 nm) in hydroponics	Microarray/qRT–PCR	922 regulated genes, e.g. superoxide dismutase (*CSD1*), copper chaperone (*CCS1*), copper transporter (*COPT2*), laccase (*LAC3*)	Landa *et al.*, (2017)
nCuO	*Lactuca sativa*	100 or 1000 mg l^–1^ CuO NP (~40-200 nm) via foliage	RNA sequencing/qRT–PCR	2270 (100 mg l^–1^ CuO NP) and 4264 (1000 mg l^–1^ CuO NP) modified genes, e.g. cellulose synthase A catalytic subunit 4 (*CESA4*), photosystem I chlorophyll *a*/*b*-binding protein 3 (*LHCA3*), chlorophyll *a*/*b*-binding protein 3C (*CAB3C*), photosystem I reaction centre subunit (*psaK*, *psaD*, *psaL*), and photosystem II reaction centre protein (*psbB* and *psbZ*) up-regulated, photosystem I chlorophyll *a*/*b*-binding protein 3 (*LHCA3*), chlorophyll *a*/*b*-binding protein 3C (*CAB3C*), photosystem I reaction centre subunit [*psaK*, *psaD* (ncbi_111898767), *psaL*], and photosystem II reaction centre protein (*psbB* and *psbZ*) down-regulated.	Xiong *et al.*, (2021)
nZnO	*Arabidopsis thaliana* Col-0, root	100 ppm ZnO NP (<100 nm) (or TiO NP (<150 nm) or fullerene soot (>7% fullerene) in hydroponics	Microarray/qRT–PCR	660 up-regulated genes (involved in abiotic and biotic stress responses), 826 down-regulated genes (involved in cell organization and biogenesis, including translation, nucleosome assembly, and microtubule-based process) by ZnO NPs.	Landa *et al.*, (2012)
nZnO	*Arabidopsis thaliana* Col-0, whole seedlings	100 or 200 mg l^–1^ ZnO NP (20-45 nm) in agar-solidified medium	RNA sequencing/qRT–PCR	A total of 1024 genes were up-regulated and 447 genes were down-regulated in nZnO-treated seedlings. After 3 d of recovery, 71% of DEGs had returned to normal levels in nZnO-treated seedlings.	Wan *et al.*, (2019)
nZnO	*Solanum lycopersicum*, leaf, root	Foliar spraying with ZnO NPs (20 and 100 mg l^–1^)	RNA sequencing/qRT–PCR	808 up-regulated and 103 down-regulated genes in ZnO NP-treated tomato leaves (e.g. nutrient, amino acid and sugar transporters, genes involved in sugar metabolism), and 395 up-regulated and 1127 down-regulated genes in tomato roots (e.g. element transporters, cutin, wax, suberin synthesis, flavonoid biosynthesis, sugar metabolism).	Sun *et al.*, (2020)
Proteomics					
nFe	*Triticum aestivum* seedlings (Pakistan-13, NARC-11 varieties), shoot	5 ppm Fe NP (20 nm) via root system	Gel-free/label-free proteomics	The abundance of proteins related to photosynthesis (e.g. ribulose bisphosphate carboxylase small chain clone 512, ribulose bisphosphate carboxylase/oxygenase activase A, phosphoglycerate kinase) and proteins involved in amino acid metabolism (e.g. ketol acid reductoisomerase, probable LL diaminopimelate aminotransferase) was decreased in the drought-tolerant variety. The abundance of proteins involved in photosynthesis (e.g. ribulose bisphosphate carboxylase small chain clone 512, phosphoglycerate kinase) was increased by Fe NP, while amino acid metabolism-related proteins (e.g. glutamate glyoxylate aminotransferase 1, ketol acid reductoisomerase) decreased in the salt-tolerant variety. Of photosynthesis-related proteins, light reaction was enhanced in the salt-tolerant variety compared with the drought-tolerant variety on exposure to Fe NPs.	Yasmeen *et al.*, (2016)
nCuO or nFeO	*Triticum aestivum* (galaxy-13, NARC-11, Pakistan-13 varieties), grain	20, 25, 35, or 40 ppm CuO NP (15–30 nm) or FeO NP (20–30 nm) in soil	Proteomics/gel-free	In 25 ppm CuO-treated galaxy-13, Pakistan-13, and NARC-11 wheat varieties, 58, 121, and 25 proteins, respectively, were changed in abundance. Glycolysis and protein degradation-related proteins were increased by both nanometals.	Yasmeen *et al.*, (2017)
nCuO	*Triticum aestivum* seedlings (Pakistan-13, NARC-11 varieties), shoot	1, 5, 10, or 50 ppm CuO NP (<50 nm) in solution	Proteomics/gel-free	Abundance of proteins related to glycolysis and the tricarboxylic acid cycle was increased by CuO NPs. Proteins related to photosynthesis and tetrapyrrole synthesis were decreased by CuO NPs in both varieties.	Yasmeen *et al.*, (2018)
Metabolomics					
nZnO	*Glycine max*, leaf, root		Metabolomics	In nZnO-exposed root, 104 changed proteins associated with secondary metabolism, cell organization, and hormone metabolism were detected. In the leaf, 16 common proteins were significantly changed in NP-exposed soybean, predominantly associated with photosystem and protein degradation.	Hossain *et al.*, (2016)
nCuO	*Arabidopsis thaliana* (Col-0), root, leaf, flowering shoot	10 mg l^–1^ CuO NP (490 ± 70 nm) in hydroponics	LC-QToF-MS and LC-Orbitrap-MS untargeted metabolomics platforms	65 metabolites altered by CuO NPs (e.g. jasmonates, scopoletin and glucosinolate derivatives). Targeted analysis: up-regulation of JA precursors (12-OPDA and dinor-12-OPDA), and down-regulation of the final product (JA).	Soria *et al*., (2019)
nMoO_3_	*Triticum aestivum*, *Zea mays*, leaf	100 or 500 mg l^–1^ MoO_3_ NPs (~375–399 nm) in vermiculite-grown plants	UHPLC, LC-MS/MS metabolomics platform	21 dysregulated metabolites in corn leaves and 53 in wheat leaves. Five more metabolomic pathways were perturbed in wheat leaves compared with corn leaves. Targeted analysis: the amounts of asparagine, fructose, reduced glutathione, and mannose were reprogrammed in both corn and wheat root and leaf.	Huang *et al.*, (2021)

## Promoting effects of essential nanometals on non-metal-accumulator plants and their practical application in plant cultivation

### Hormetic effects of essential nanometals on germination, vegetative growth, and yield

Since seed germination is an early and sensitive phase of plant ontogenesis, it is important to determine the influencing effects of different nanomaterials (including essential nanometals) on it, especially in crops. Thus, the concentration-dependent hormetic effect of essential nanometals on germination and seedling development has been revealed by several authors in the past years, and has been recently reviewed by Szőllősi *et al.* (2020). More recent literature shows a trend for authors to prefer biological, plant-based NP production over chemical synthesis. For example, ultra-small (~6.6 nm) CuO NPs derived from plant-based synthesis (green tea extract) at a lower concentration positively affect the germination and seedling growth of lettuce, whereas elevated doses of nano CuO (nCuO; hereafter, nanoforms of essential metals and their compounds are indicated with the prefix n) have an adverse effect. The toxicity of CuO NPs was shown to be associated with inhibited antioxidant defence and intensified *S*-nitrosothiol signalling (Pelegrino *et al.*, 2020). Similarly, plant-extract-derived, phytochemical-capped FeO NPs were used to improve the germination capacity of rice (*Oryza sativa* L.), whereas the same concentrations of the ionic form (FeSO_4_) had an inhibitory effect (Afzal *et al.*, 2021). The germination-promoting effect of nFeO was due to the enhancement of water uptake, alpha-amylase activity, soluble sugar content, and antioxidant enzyme activities. The observed nFeO-induced ROS production may be involved in cell wall loosening and endosperm weakening. Moreover, the contents of macro- and micronutrients in the endosperm of the nFeO-treated rice seeds increased, supporting that FeO NPs can be used effectively as a ‘nano-nutrient’ for efficient germination and growth (Afzal *et al.*, 2021). In another recent study, MnO NPs were synthesized using onion (*Allium cepa* L.) bulb extract and were applied for seed priming of watermelon (*Citrullus lanatus* L.) genotypes (Kasote *et al.*, 2021). In comparison with the bulk forms (KMnO_4_ and Mn_2_O_3_), the seed treatment with nMnO proved to be less toxic. Changes in the leaf metabolome of seedlings showed a clear MnO NP effect only in case of the triploid line. Furthermore, the levels of phytohormones such as abscisic acid, gibberellic acid, salicylic acid, and JA changed in a genotype-dependent manner in MnO NP-treated watermelon, which emphasizes the genotype dependence of plant responses to nanometals (Kasote *et al.*, 2021). Similarly, the effect of nFe and nCu on seedling development varied depending on the genotype of Scots pine (*Pinus sylvestris* L.) (Kalyakina *et al.*, 2021). A hormetic effect of Mo NPs on seedling development has been described, without a significant effect on germination of rice (Adhikari *et al.*, 2013). In a recent study, rice seedlings were treated with high doses of chemically synthesized Mo NPs (α-MoO_3_ and MoS_2_) and decreases in pigment content and antioxidant activities were described, indicating Mo NP-induced oxidative stress in rice seedlings. Furthermore, the authors concluded that, based on the poor translocation and low accumulation without any notable effect on seedling growth, a concentration of MoS_2_ NPs of 100 ppm appears to be a promising treatment for future application during rice cultivation (Sharma *et al.*, 2021).

Far fewer studies have dealt with the later developmental stages of plants, for example, flowering, and yield in connection to essential nanometals. A study found that treatment of soybean seeds with Fe, Cu, or Co NPs (zerovalent) slightly induced germination and, when used in a field experiment, increased chlorophyll content and other parameters such as number of nodules per root, number of pods per plant, pod weight, and 1000-grain weight; nCo exerted the most efficient promoting effect, followed by Cu and Fe NPs (Ngo *et al.*, 2014). In carrot, the highest rate of root and shoot growth induction and yield was achieved by a combined treatment with ZnO and FeO NPs (Elizabath *et al.*, 2017). In a recent field study, the combined foliar application of ZnSO_4_ and nZnO produced the highest yield and improved nutrient content of rice compared with ZnSO_4_-fertilized plants, indicating that the application of ZnO NPs as additives in fertilizers is beneficial in agricultural practice (Elshayb *et al.*, 2021). Similarly, the application of nFe on greenhouse-grown tomato significantly enhanced growth and yield, and the promoting effect of nFe proved to be more intense than that of conventional Fe fertilizers (El-Desouky *et al.*, 2021).

Most of the studies on the effects of essential nanometals on plant yield are descriptive, without revealing the biochemical and molecular mechanisms explaining the observed effects. Future studies should include an omics approach in order to understand the responses of developing plants to essential nanometals at different levels.

Seed priming by essential nanometals for inducing germination capacity and vegetative and reproductive growth seems to be a promising way to increase the effectiveness (in terms of cost, time, quality, and quantity) of crop cultivation. The effects, however, depend on several factors, making the development of general treatment protocols difficult. In the case of seed nanopriming, the use of plant-synthesized NPs has become more and more desirable; thus, ‘green nanopriming’ will surely be a focus of future research (Song and He, 2021).

### Essential nanometals induce phytochemical synthesis in plants

Beyond affecting primary metabolism and consequently promoting growth and development, nanometal treatments elicit the production of secondary metabolites in diverse plant species. More than 5000 phytochemicals have been identified in fruits, vegetables, and grains. Among the most valuable phytochemicals are vitamin C, folate, provitamin A, potassium (K), calcium (Ca), Mg, flavonoids, phenolic acids, alkaloids, carotenoids and fibres, which have a wide range of applications in cosmetics, the food industry, pharmacology, and medicine due to their beneficial effects. According to the very recent review of Rivero-Montejo *et al.* (2021), among the essential nanometals, mostly nCu and nZn as biostimulants have been examined so far. Nanostimulation by the application of Cu or Zn NPs increases the total phenol, flavonoid, rebaudioside A, stevioside, thymol, and carvacrol contents of diverse plant species (Juarez-Maldonado *et al.*, 2018, Rivero-Montejo *et al.*, 2021). More recently, some studies have applied essential nanometals as biostimulants in plants exposed to abiotic stress. For example, foliar application of ZnO NP on gold-of-pleasure plant (*Camelina sativa L.*) exposed to salt stress increased the total phenol content and the levels of anthocyanins, carotenoids, Ca, Zn, and phosphorus (P) compared with plants exposed to salt stress alone (Hezaveh *et al.*, 2020). In another study, bell pepper (*Capsicum annuum* L.) exposed to salt stress was treated with selenium (Se), silicon (Si), or Cu NPs, and in each case it was found that the NPs stimulated ascorbate peroxidase, glutathione peroxidase, catalase, and phenylalanine ammonia lyase activities and increased the content of phenols, flavonoids, glutathione, β-carotene, and yellow carotenoids in the fruits, suggesting that the application of Se, Si, or Cu NPs to bell pepper plants during conditions of high salinity is an efficient way to increase the content of bioactive compounds in fruits (González-Garcia *et al*., 2021).

For this subfield, in addition to observing changes in compound composition, it is also necessary to understand the molecular mechanisms behind the effects of essential nanometals. Nevertheless, nanobiostimulation is a promising approach because low doses of NPs can trigger the accumulation of beneficial plant compounds, thus making cultivation cost-effective. As during nanostimulation nanometals may be introduced directly into products for human consumption, further efforts should be made to assess the toxicological risks.

### Essential nanometals intensify biotic and abiotic stress tolerance in plants: focusing on nutrient deficiency

In association with global climate change and with the increasing consumption of plant-based food, it is becoming increasingly important to intensify the resilience of (crop) plants to environmental stresses (Kilian *et al.*, 2021). The living environment (biotic factors) exerts notable impacts on the physiological processes of plants. Viruses, viroids, phytoplasma, bacteria, fungi, oomycetes, nematodes, insects, and weeds cause major problems in crop production. According to certain estimates, yield losses of major crops due to pests range from 20% to 40% or even more than 50% in certain cases (Pestovsky and Martinez-Antonio, 2017), causing considerable financial damage for farmers (Hajong *et al.*, 2019). Therefore, the introduction of new approaches such as the utilization of nanomaterials, including nanometals, in agricultural practices is urgently needed. Crop yield loss can be reduced by detecting infections as soon as possible; thus, an important area is the development and application of NPs as sensors in crop production (Li *et al.*, 2020). Among the nanometals, mostly nCuO, nZnO, nFe_2_O_3_, nFe_3_O_4_, and nNiO have potential as nanopesticides in disease management practices against viruses, bacteria, fungi, and oomycetes. The current interest and significance of this topic is indicated by the large number of detailed literature reviews that have been published recently (e.g. Djiwanti and Kausik, 2019; Hajong *et al.*, 2019, de Oliveira, 2021; Farooq *et al.*, 2021).

Besides biotic factors, non-living environmental conditions (abiotic stress factors), such as water or nutrient limitations, heat, cold, salinity, and UV radiation, reduce the average yield of most major crop plants by more than 50% (Hirayama and Shinozaki, 2010). Based on the experimental data accumulated in the past years, it can be stated that nanomaterials, including nanometals, provide a promising strategy to address these environmental challenges that plants face. This topic is a focus of several recent reviews (Kahn and Upadhyaya, 2019; Zhao *et al.*, 2020; Singh *et al.*, 2021; Thounaojam *et al.*, 2021).

One of the significant abiotic stressors influencing crop quantity and quality is the lack or inadequate accessibility of essential nutrient elements in soils. Plants require optimal availability of at least 14 macro- (N, P, K, S, Ca, Mg) and micronutrients (Cu, Zn, Mn, B, Mo, Si, Ni, and Cl) (Kirkby, 2012). Particularly in developing countries, low soil fertility, reduced availability of mineral nutrients in soils, improper nutrient management strategies, and the lack of plant genotypes possessing tolerance to nutrient limitations collectively lead to food insecurity, malnutrition, and degradation of the ecosystem. About 50% of the world’s population is endangered by an inadequate micronutrient supply, highlighting the necessity of implementing new strategies (Kumari *et al.*, 2021).

Plant breeding is a good option to develop plant genotypes with improved potential to acclimatize to nutrient-deficient conditions (Kumari *et al.*, 2021). Another option is to increase the available element content of the soil by fertilization; however, the application of conventional fertilizers has multiple drawbacks. Most importantly, the nutrient use efficiencies of conventional fertilizers are only around 30–35% for N, 18–20% for P, and 35–40% for K, meaning that the applied nutrients are utilized by plants with low efficiency. Moreover, the large-scale application of conventional fertilizers causes irreversible damage to the soil structure, mineral cycles, and soil microbiome, as well as to plants (Mohammadi and Sohrabi, 2012; Solanki *et al.*, 2015; Riaz *et al.*, 2020). Nanofertilizers release nutrients slowly and steadily, providing balanced nutrition for crops without causing damage to soils, microbes, or plants (Elemike *et al.*, 2019; Mittal *et al.*, 2020). Using nanoforms of fertilizers reduces nutrient loss from the soils; thus, this is an ‘eco-friendly’ way of land use that supports sustainable agriculture (Subramanian *et al.*, 2015). However, despite the significant practical potential of nanofertilizers, the amount of laboratory data about the effects of NPs and nanometals on element-deficient plants is surprisingly low.

Inadequate Zn availability for crops is a relatively common problem worldwide. Bala *et al.* (2019) applied ZnO NP treatments (at 0.5, 1, or 5 g l^–1^) to the leaves of rice grown in soil with adequate or low Zn supply. The ZnO NPs could be detected on the leaf surface near to the stomata by SEM, indicating that one putative route for internalization of NPs is via stomata. The foliar application of ZnO (5 g l^–1^) has positive effect on the soil microbiome, possibly due to the increased Zn ion concentration of the roots. For the Zn-deficient plants, ZnO NP treatment improved vegetative growth and yield parameters (e.g. tiller number, 1000-grain weight), enhanced Zn content, and altered levels of nutrients in the root, shoot, and grain. These results indicate that foliar nZnO fertilization successfully reverts the symptoms of Zn deficiency in rice (Bala *et al.*, 2019).

The recent work of Sun *et al.* (2020) revealed that the application of nZnO to tomato plants through the foliage increases Fe accumulation and efficiently improves tolerance of Fe deficiency due to decreasing Fe-deficiency-induced oxidative damage and enhancing the contents of essential metal elements. Additionally, transcriptomic and metabolomic analyses indicated that the expression of genes involved in the antioxidant system, transport, carbon and N metabolism, and secondary metabolism was increased by ZnO NP spraying, possibly contributing to the Fe-deficiency tolerance of tomato (Sun *et al.*, 2020).

The efficiency of nCuO as a fertilizer has also been studied; however, those experiments did not involve element-deficient plants. Soil-grown Chinese scallions (*Allium fistulosum* L.) were treated with nCuO, bulk CuO, and Cu in ionic form (CuSO_4_). The nanoform of CuO proved to be the most efficient in increasing the contents of Ca, Fe, or Mg, as well as allicin (Wang *et al.*, 2020). Similar results were obtained by Kohatsu *et al.* (2021) in a study in which the foliar application of biogenic CuO NPs triggered the accumulation of K, Na, S, Ag, Cd, Cr, Cu, and Zn in lettuce leaves without causing necrosis, while CuSO_4_ caused the most intense changes in the mineral element content of lettuce and caused necrotic damage. These findings indicate that nCuO is a more effective fertilizer than the other forms of Cu for lettuce cultivation (Kohatsu *et al.*, 2021).

Peanut (*Arachis hypogaea* L.) is sensitive to limited Fe supply, and therefore nFe fertilizers have great potential for this crop. In the study of Rui *et al.* (2016), Fe_2_O_3_ NPs increased growth and biomass production, and the SPAD values (a measure of chlorophyll content) of peanut plants. Moreover, nFe_2_O_3_-induced changes in the levels of certain phytohormones [abscisic acid, gibberellic acid (GA_4 + 7_, GA_3_), zeatin riboside, dihydrozeatin riboside] and antioxidant activities (peroxidase, SOD, catalase) were associated with the growth induction. Additionally, nFe treatment efficiently increased the Fe content of peanut plants, indicating that nFe_2_O_3_ is a promising nanonutrient for peanut plants, although the putative amelioration of damage in Fe-deficient plants would have provided additional relevant data (Rui *et al.*, 2016).

Not all types of nFe can be effective as a source of Fe for plants. A recent study indicated that despite the fact that supermagnetic iron oxide NPs (SPIONs, ~12.5 nm) internalize in the roots, this treatment alone is not effective in supplying Fe to summer squash (*Cucurbita pepo* L.); however, the authors suggest that applying SPIONs together with Fe(III-EDTA) can be beneficial in the fertilization of *Cucurbita* spp. (Tombuloglu *et al.*, 2019).

The phyto-effect of nFe also depends on the plant species and their Fe-acquisition strategies. Both cucumber and maize used Fe and P derived from FePO_4_ NPs more efficiently than when supplied as bulk material, but cucumber mainly used FePO_4_ NPs as a source of P, while maize preferred FePO_4_ NPs as a source of Fe. Interestingly, cucumber roots were not able to take up the nFe, possibly due to agglomeration. These comparative results clearly indicate that the nutrient utilization of roots exposed to FeSO_4_ NPs is affected by species-specific metabolic responses (Sega *et al.*, 2020).

It is also true for NPs studied as nanofertilizers that the effects on the plant may differ depending on the method of application. One of the few studies examining nMn found that foliar exposure resulted in higher shoot and grain Mn contents, lower soil nitrate-N, and higher soil and shoot P compared with soil application of nMn in wheat plants. These results indicate that applying nMn via the foliage can exert more intense effects on nutrient composition of wheat. Additionally, exposure to the nanoform of Mn via the soil could mildly affect plants, differing from exposure to bulk or ionic Mn (Dimpka *et al*., 2018).

The above examples show that, with few exceptions, essential nanometals are better utilized by plants and have a more positive effect on plant biomass production, as well as more efficiently alleviating nutrient deficiency, than bulk materials. One of the reasons behind this could be that due to their nanometric size, a greater amount of NPs may reach the root surface compared with the bulk forms. Moreover, NPs may be more rapidly and more efficiently dissolved due to their large surface-to-volume ratio. Additionally, the literature clearly points out that the beneficial effects of essential nanometals depend on factors such as the form, concentration, and route of administration. Furthermore, the plant species and its element acquisition strategy may influence the observed effects of nanofertilizers (Kalwani *et al.*, 2022).

## Conclusions and future perspectives

Plants use Cu, Fe, Mo, Mn, Zn, and Ni as essential metals for operating vital physiological processes, such as photosynthesis, cellular respiration, and antioxidant protection. Nanoforms of these metals possess a number of favourable properties over bulk materials, such as a large surface area and superior electrical, optical, magnetic, and biological properties.

Essential nanometals come into contact with plant leaves and roots and enter the tissues through diverse pathways. In the case of roots, soil microbes and root exudates may help or limit NP uptake, while in the case of leaves opened stomata or thin permeable regions of the epidermis can serve as entry points for NPs. At the cellular level, the movement of NPs is limited by the cell wall pore size and the Casparian strip, and the uptake of NPs into the cytoplasm may occur by endocytosis or interaction with carriers and channels. Between the cytoplasm of adjacent cells, NPs may travel through the plasmodesmata.

The detection of nanometals in plant tissues faces technical difficulties, so it is recommended to use several complementary methods (e.g. TEM, ICP-MS). Biotransformation of nanometals may take place in the plant tissues, with the ionic form of the metal being released from the nanometal taken up by the plant. Our knowledge about the internalization, behaviour, and interactions of essential nanometals in plant cells is incomplete, so more studies are needed to examine the transport mechanisms and biomolecular interactions from a physico-chemical point of view.

A more actively studied topic is the effect of essential nanometals on plants, which is concentration dependent and can therefore be considered as hormesis. Metal ions also exert hormetic effects on plants, but in the case of nanometals there are some special influencing factors, for example, the NP size. Furthermore, the physico-chemical properties of the nanometals, the properties of the plant species, and the conditions of the plant treatments affect the hormetic effect. All of these factors indicate that the effects of essential nanometals on plants can be highly variable among experimental systems, which makes it difficult to draw general conclusions and points out the necessity of further tests in different experimental setups. To get a holistic view, plant responses need to be examined with (multi)omics approaches. From the data available so far for nZn, nFe, nCu, and nMo, a picture is beginning to emerge that processes mainly involved in stress responses are activated at the genome, proteome, and metabolome levels, with more significant changes in the root than in the shoot. These data should be completed by the examination of nMn and nNi using (multi)omics approaches.

The beneficial effects of essential nanometals include promoting germination and vegetative and reproductive development, promoting the synthesis of phytochemicals (e.g. secondary metabolites), and enhancing abiotic and biotic stress tolerance. In most cases, laboratory or field studies do not cover the molecular details, and thus plant physiological responses need to be examined in more depth in the future.

The multifaceted effects of essential nanometals provide the basis for their application in plant cultivation as priming agents, nanofertilizers, nanopesticides, or nanoherbicides. This review has focused on the effects of nanometals in ameliorating nutrient deficiency in plants. In many cases, the effects of nanometals as nanofertilizers are tested on healthy plants, with far fewer studies dealing with amelioration of metal deficiency, which would, however, be required. The use of essential nanometals in sustainable agriculture is a desirable strategy, as efficient cultivation can be achieved with less environmental impact. Also fitting into the environmentally friendly view, the biogenic approaches utilizing microbe or plant sources for producing NPs have become increasingly popular in recent studies. At the same time, we should consider the risks (both environment- and health-related) during the application of nanomaterials. Although we are still in the early stages of understanding the molecular-level responses of plants to essential nanometals, we can state that the effects exerted by essential nanometals are more beneficial than those of the bulk forms, indicating the potential of their application in future agricultural practices.
